# Effects of dietary protein levels on production performance, meat quality and flavor of fattening pigs

**DOI:** 10.3389/fnut.2022.910519

**Published:** 2022-07-22

**Authors:** Dong Wang, Guoshun Chen, Minjie Chai, Chengrui Shi, Yiwen Geng, Yuyan Che, Yancui Li, Shuaishuai Liu, Yancheng Gao, Haoxia Hou

**Affiliations:** ^1^College of Animal Science and Technology, Gansu Agricultural University, Lanzhou, China; ^2^Pingliang Animal Husbandry and Fishery Station, Pingliang, China; ^3^College of Food Science and Engineering, Gansu Agricultural University, Lanzhou, China; ^4^Gansu Longyuan Agricultural Economic Cooperation Center, Lanzhou, China

**Keywords:** dietary protein level, finishing pig, production performance, meat flavor, meat quality

## Abstract

This study aimed to evaluate the effects of dietary protein level on the production performance, slaughter performance, meat quality, and flavor of finishing pigs. Twenty-seven Duroc♂ × Bamei♀ binary cross-bred pigs (60.86 ± 2.52 kg body weight) were randomly assigned to three groups, each group has three replicates, and each replicate has three pigs. Three groups of finishing pigs were fed 16.0, 14.0, and 12.0% crude protein levels diets, and these low-protein diets were supplemented with four limiting amino acids (lysine, methionine, threonine and tryptophan). The results showed that the pigs fed low-protein diets increased *(P* < 0.05) loin eye muscle area, and reduced (*P* < 0.05) heart weight, lung weight. The feed-weight ratio of the 14.0% protein group was reduced (*P* > 0.05); Dietary protein levels significantly affected the luminance (L_24h_), yellowness (b_45min_ and b_24h_) (*P* < 0.05), reduced shear stress, muscle water loss, drip loss, the levels of crude fat (*P* < 0.05), and increased marbling score (*P* < 0.05) in the muscle of finishing pigs; The low-protein diets improved PUFA/TFA, PUFA/SFA (*P* > 0.05), and increased hexanal, E-2-heptenal, 1-octen-3-ol, EAA/TAA in the muscle of finishing pigs (*P* < 0.05); The results indicated that reduced the crude protein levels of dietary by 2.0–4.0%, and supplementation with four balanced limiting amino acids had no significant effects on the production performance and slaughter performance of finishing pigs, and could effectively improve meat quality and flavor.

## Introduction

Low protein amino acid balanced diet can meet the needs of livestock and poultry by reducing the protein level in the diet and adding free amino acids. It can improve the utilization rate of livestock and poultry feed protein, reduce production costs and reduce environmental pressure. Its application in the breeding industry is of great significance. Adding industrial synthetic amino acids to feed to appropriately reduce dietary protein levels can effectively reduce feed costs, regulate gut microbiota structure, improve gut morphology, increase nitrogen utilization, reduce harmful gas emissions (ammonia), and can Improve gut health without compromising pig performance (1). Traditional corn-soybean meal diets typically have increased levels of soybean meal to meet the lysine requirements of pigs. This leads to excessively high dietary protein levels and low protein utilization. At the same time, animal undigested protein is excreted in large quantities through feces and urine, causing serious environmental pollution (2).

In pig production, when the requirements of essential amino acids (EAA) and total nitrogen are met, the levels of crude protein in the diet could be reduced as protein requirements in pigs are essentially those of amino acids pig educed normal protein levels by 4% and balanced the dietary levels of EAA (3). Wang et al. found that a low-protein (13.5% CP) diet had no significant effect on growth performance, but significantly improved apparent nitrogen digestibility and nitrogen deposition rate, and significantly reduced nitrogen emissions in manure and urine in finishing pigs (4). Xu et al. found that neither a low-protein diet nor a high-protein diet had any significant effects on the backfat thickness and loin eye muscle area in finishing pigs, the low protein group has an increasing trend (5). Li et al. found that feeding a low-protein diet significantly increased the redness of muscle in finishing pigs, whereas feeding low-protein and very low-protein diets significantly reduced muscle shear force (6). Furthermore, low-protein diets significantly improved the levels of intramuscular fat and monounsaturated fatty acids (MUFA) in the muscle of finishing pigs. Addition of alpha ketoglutarate to low-protein diets significantly increased the levels of intramuscular fat, oleic acid, and MUFA in the muscles of finishing pigs (7).

Current literature on low-protein diets of finishing pigs has been focused mostly on growth performance, carcass traits, and meat quality indicators. However, only a few studies have evaluated the effects of low-protein, amino acid balanced diets on volatile compounds in the muscle of finishing pigs. Early research by our group found that reducing the dietary protein level of Du × Min crossbred finishing pigs can significantly affect the growth performance and slaughter performance, and have positive effects on improving meat quality and muscle nutrient composition (8). On the basis of previous study, this study evaluated the effects of various low-protein, amino acid balanced diets on production performance, slaughter performance, meat quality, and flavor in finishing pigs. The results provide a theoretical basis for the application of a low-protein, amino acid balanced diet in pig production.

## Materials and methods

### Ethics statement

Experiments involving animals were carried out in accordance with regulations for the Administration of Affairs Concerning Experimental Animals (Ministry of Science and Technology, China; revised in June 2004). Sample collection was carried out according to the guidelines of the Ethics Committee for the Care and Use of Laboratory Animals of Gansu Agricultural University.

### Experimental design and feeding management

The experimental pigs were all selected from the parent-generation pig farm of Gansu Agriculture and Animal Husbandry Fine Breeding Farm (Jingtai County, Gansu Province). The experimental animals were Duroc ♂ × Bamei ♀ binary cross-bred pigs that were close in parity, age, health status, and initial weight.

A total of 27 Duroc heads were selected for the test ♂ × Bamei ♀ Binary cross bred pigs, which is 120 days old and weighs 60.86 ± 2.52 kg, and similar health status. They were randomly divided into the control group, group I, and group II. There were three replicates in each group and three pigs in each replicate. Conduct 5-day pre-test, and the formal test period is 60 days. The details of each experimental group are presented in Table 1.

**Table 1:** Study design.

**Items**	**Control**	**Group**	**Group**
	**group**	**I**	**II**
Dietary crude protein levels/%	16.0	14.0	12.0

### Dietary composition and nutrition level

The reference index for finishing pigs with a body weight of 60.86 ± 2.52 kg was selected, based on the low-protein dietary recommendations in the “Piglet, Growing and Finishing Pig Compound Feed” standard, proposed by the China Feed Industry Association Group Standards Technical Committee in 2018. These recommendations were combined with the methods of Bai (9). Based on previously published data on low-protein diets with standardized ileal digestibility and supplemented amino acids; we designed three diets with crude protein levels of 16.0, 14.0, and 12.0%, respectively. The composition and nutritional level of the basic diets for the experimental pigs are shown in Table 2.

**Table 2:** Composition and nutrient levels of experimental diets (air-dry basis).

	**Control group**	**Group I**	**Group II**
Ingredients			
Corn/%	63.5	68	72
Soybean meal/%	18.3	12.9	7.2
Wheat bran/%	5	5	5
Alfalfa meal/%	5	6.5	9
Bentonite/%	4	2.9	1.6
Soybean oil/%	1.5	1.7	2
Compound enzyme/%	0.1	0.1	0.1
Lysine/%	0.09	0.23	0.37
Methionine /%	–	0.03	0.05
Threonine /%	–	0.08	0.15
Tryptophan /%	–	0.03	0.05
CaCO_3_/%	0.51	0.46	0.3
CaHPO_4_/%	1.15	1.22	1.33
Premix^a^/%	0.5	0.5	0.5
NaCl/%	0.35	0.35	0.35
Total/%	100	100	100
Nutrient levels^b^			
DE/MJ/kg	13.14	13.13	13.11
CP/%	15.94 (16.00%)^c^	14.00 (14.00%)^c^	12.05 (12.00%)^c^
CF/%	3.22	3.5	4.02
Ca/%	0.6	0.61	0.6
TP/%	0.55	0.55	0.55
Na/%	0.16	0.16	0.17
Cl/%	0.27	0.27	0.28
SID Lysine/%	0.86	0.86	0.86
SID Methionine/%	0.26	0.26	0.26
SID Threonine/%	0.59	0.59	0.59
SID Tryptophan/%	0.19	0.19	0.19

a *The premix provided the following per kg of the diet: Fe 64.00 mg; Zn 71.00 mg; Mn 35.00 mg; Cu 17.00 mg; Se 0.36 mg; I 0.64 mg; vitamin A 790 IU; vitamin D3 135 IU; vitamin E 55.00 mg; thiamine (vitamin B1) 2.20 mg; riboflavin (vitamin B2) 2.50 mg; biotin 0.05 mg; folic acid 0.35 mg; nicotinic acid 29.00 mg; calcium pantothenate 27.00 mg; vitamin B6 0.09 mg; vitamin B12 1.00 mg; choline chloride 5000.00 mg; flavor 3000.00 mg; sweeteners 3000.00 mg; phytase 4000.00 mg; lysine 30000.00 mg; tryptophan 2000.00 mg*.

b *Nutrient levels were calculated*.

c *Set experimental values in parentheses*.

*SID, standardized ileal digestibility*.

### Feeding management

The pigs were raised on the parent-generation pig farm of an agricultural and animal husbandry breeding farm in Gansu Province. The pig house was cleaned and disinfected before the study. During the study, pigs were dewormed and immunized, according to standard pig farm management protocols. The pigs had free access to feed and water. The pig house cleaned twice daily and disinfected once a week. During the test period, the amount of feed given and the amount remaining in each group were recorded every day, and the daily feed intake was calculated. After the experimental period, each pig was weighed on an empty stomach.

## Measurement indicators and methods

### Growth performance

During the trial period, pigs were weighed before morning feeding and at the beginning and end of the trial. Body weight at the beginning and end of the trial were recorded, and the average daily weight gain was calculated. Furthermore, the daily feed volume and the remaining volume in the feed trough were recorded, and the average daily feed intake was calculated. The feed-to-weight ratio was calculated, based on the average daily gain and average daily feed intake, according to the following formulas:


$$\begin{array}{r} \text{ ADG }=\;\left(\text{final weight }-\text{ initial weight}\right)/\text{no of days fed} \end{array}$$

$$\begin{array}{r} \text{ADFI }=\text{ TFC}/\left(\text{feeding days ×number of feeding}\right) \end{array}$$

$$\begin{array}{r} \text{Ratio of Feed to Weight }=\text{ADFI}/\text{ADG}\times 100\% \end{array}$$

ADG represents Average daily gain. ADFI is Average daily feed intake.

### Slaughter performance

Before slaughter, pigs were fasted for 24 h and allowed to drink water freely. One pig was randomly selected from each repetition in each group, to be weighed and slaughtered. Three pigs were slaughtered in each group; thus, a total of nine pigs were slaughtered. Slaughtering and sampling were carried out in strict accordance with the “Good Practice for the Slaughtering of Livestock and Poultry - Pigs” (Chinese Standard GB/T 19479-2019). The warm carcass was weighed, and the carcass weight to live body weight ratio was considered to represent the slaughter rate.

The average thickness of back-fat was measured, according to the Chinese “Feeding standard of swine” (Chinese Standard NY/T 65 - 2004). The loin eye muscle area was measured as follows: the contour of the loin eye muscle was drawn on sulfite paper with a pencil, and the height and width of the muscle was measured with vernier caliper The area of the loin eye muscle was then calculated according to the following formula: height × width ×0.7 (10). The oblique length of the carcass was measured as follows: a hook was placed into the left hock of the carcass, which was then hung upside down. The length from the leading edge of the pubic symphysis to the leading edge of the first rib and sternum was measured with a meter rule, Meat quality analysis was also performed, and the levels of inosinic acid, amino acids, fatty acids, and volatile flavor substances were evaluated in samples of the longissimus dorsi between the first and second ribs (11).

### Meat quality measurements

The shear force tenderness was determined according to the methods outlined in “The Determination of Shear Force for Meat Tenderness” (Chinese Standard NY/T 1180-2006). Water loss rate (hydraulic) was determined according to the methods outlined in the “Determination of Meat Quality of Livestock and Poultry” (Chinese Standard NY/T 1333-2007). Cooking loss, marbling, and pH were determined by standard procedures, previously published (12). Meat color was determined by a CR-10 meat color tester.

### Muscle nutrient content

The moisture content was determined by the electric oven drying method at 105°C (Chinese Standard GB 5009.3-2016). The crude ash content was determined using a muffle furnace at 550°C (Chinese Standard GB 5009.4-2016). The crude protein content was determined by the semi-trace Kjeldahl method (FOSS Kjeldahl apparatus, Chinese Standard GB 5009.5-2016). The crude fat content was determined by the Soxhlet extraction method (Chinese Standard GB 5009.6-2016).

### Muscle inosinic acid content

High performance liquid chromatography was used to evaluate inosinic acid levels (13), under the following conditions: a C18 column (4.6 ×250 mm, 5 μm) was used with a 260 nm UV detector. The column temperature was 25°C; the injection volume was 10 μL; the flow rate was 1 mL/min, and the retention time was 35 min.

### Amino acid contents in muscles

The levels of 17 amino acids were determined according to the methods outlined in the “Determination of Amino Acids in Food” (Chinese Standard GB/T 5009.124-2003).

### Content of fatty acids in muscles

The levels of each fatty acid were determined according to the methods outlined in the “Determination of Fatty Acids in Food” (Chinese Standard GB 5009.168-2016).

### Volatile compounds in muscles

The volatile substances in fresh meat were separated and identified by gas chromatography-massspectrometry (GC-MS) on an Agilent GC analyzer. Samples were pretreated as follows: 3 g of each specimen was placed into 20 mL bottles (no more than 1/4 of the capacity of the bottle); the bottle was capped, and heated for 40 min at 100°C. A solid-phase microextraction needle was then used for a total of 30 min for extraction and manual sampling (This was followed by agitation at 250°C for 10 min; cooling to 20°C; and successive washing with methanol, ethanol, ether, n-hexane, deionized water, and again with methanol). During extraction, the needle remained in the injection port for 5 min (14).

The gas phase conditions were as follows: an hP-5 ms GC column was used (30 m ×0.25 mm ×0.25 μm). Helium was used as a carrier gas at a flow rate of 1.0 mL/min without diversion. The inlet temperature was 250°C, and the column temperature was 40°C. The initial temperature was set at 35°C for 2 min, after which the temperature was increased to 230°C, at a rate of 5°C/min, and maintained for 5 min (15).

The conditions for mass spectrometry were as follows: electron ionization source energy, 70 eV; and doubling voltage 1,400 V. The temperature of the ion source and interface was 250°C, and the scanning mass range (M/Z) was = 20–500, with an interval of 0.3 s (16).

### Data processing and analysis

The Excel 2016 software was used for preliminary statistics and sorting of experimental data, followed by the SPSS 22.0 software for further analysis. One-way anova analysis of variance was evaluated, and the Tukey's multiple range test was used to assess significant differences between various pairs of means. *P* < 0.05 was considered significant. The results were all expressed as the mean ± standard deviation (SD).

## Results

### Effects of dietary protein level on growth performance of finishing pigs

Compared with the control group (fed 16% crude protein), the ADFI of the 12% protein group was reduced by 4.85% (*P* > 0.05) (Table 3). The ADG in the 14% protein group was increased by 2.22% (*P* > 0.05), and that in the 12% protein group was reduced by 6.67% (*P* > 0.05). The ratio of feed to weight in group I was 2.34% lower (*P* > 0.05). Thus, a low-protein diet with a crude protein level of 14.0% could increase the ADG, reduce the feed-weight ratio, and improve the production performance of finishing pigs.

**Table 3:** Effect of dietary protein level on growth performance in finishing pigs.

**Items**	**Control group**	**Group I**	**Group II**	***P*-value**
Initial BW/kg	61.26 ± 3.27	60.79 ± 2.94	60.53 ± 2.39	0.877
Final BW/kg	119.44 ± 4.39	120.81 ± 6.56	115.48 ± 5.62	0.522
ADFI / kg∙day-1	2.68 ± 0.13	2.70 ± 0.16	2.55 ± 0.12	0.412
ADG /kg∙day-1	0.90 ± 0.02	0.92 ± 0.06	0.84 ± 0.05	0.171
Ratio of feed to gain(F/G)	2.99 ± 0.09	2.92 ± 0.02	3.02 ± 0.07	0.238

*BW, body weight. Values in the same row, either without letter superscripts or the same letter superscripts indicate no significant difference (P >0.05). Different letter superscripts indicate significant differences (P < 0.05). Crude protein levels of 16.0, 14.0, and 12.0% were fed to the control group, group I, and group II, respectively*.

### Effects of dietary protein levels on slaughter performance, organ weight, and intestinal pH in finishing pigs

Compared with the control group, the average thickness of back-fat of the 14 and 12% protein groups were reduced by 1.49 and 13.82%, respectively (*P* > 0.05) (Table 4). Furthermore, skin thickness was reduced by 9.70 and 9.03% in the 14 and 12% protein groups, respectively (*P* > 0.05). The area of the loin eye muscle was increased by 20.03% in the 14% protein group (*P* < 0.05%), and increased by 14.88% (*P* > 0.05%) in the 12% protein group. The heart weight in the 14% and 12% protein groups were reduced by 18.87 and 24.53%, respectively (*P* < 0.05), and the lung weight was reduced by 25.88% (*P* < 0.05) and 3.53% (*P* > 0.05), respectively.

**Table 4:** Effect of dietary protein level on slaughter performance, organ weight, and gastrointestinal tract pH of finishing pigs.

	**Control group**	**Group I**	**Group II**	***P*** **-value**
Carcass weight/kg	88.35 ± 4.52	90.67 ± 6.41	85.57 ± 4.15	0.522
Dressing percentage/%	73.95 ± 1.05	75.01 ± 1.30	74.09 ± 0.17	0.413
Backfat thickness/mm	26.92 ± 3.10	26.52 ± 1.49	23.20 ± 6.01	0.501
Skin thickness/mm	2.99 ± 0.11	2.70 ± 0.70	2.72 ± 0.09	0.647
Carcass length/cm	105.62 ± 3.72	107.18 ± 1.00	102.03 ± 6.29	0.377
Loin eye area/cm^2^	38.04 ± 4.56^b^	46.04 ± 1.95^a^	43.70 ± 1.00^ab^	0.034
Hind legs proportion/%	15.50 ± 0.22	14.82 ± 0.90	14.91 ± 0.30	0.341
Heart weight/kg	0.53 ± 0.03^a^	0.43 ± 0.06^b^	0.40 ± 0.00^b^	0.011
Liver weight/kg	1.68 ± 0.43	1.80 ± 0.61	1.95 ± 0.15	0.761
Spleen weight/kg	0.23 ± 0.03	0.22 ± 0.03	0.20 ± 0.00	0.379
Lung weight/kg	0.85 ± 0.10^a^	0.63 ± 0.08^b^	0.82 ± 0.08^a^	0.039
Kidney weight/kg	0.45 ± 0.00	0.35 ± 0.09	0.35 ± 0.05	0.131
Stomach weight/kg	0.98 ± 0.08	1.10 ± 0.15	0.92 ± 0.03	0.139
Gastric contents pH	3.66 ± 0.42	3.73 ± 0.22	3.81 ± 0.23	0.827
Cecal contents pH	5.78 ± 0.14	5.44 ± 0.18	5.53 ± 0.22	0.138
Jejunal contents pH	5.65 ± 0.08	5.65 ± 0.13	5.62 ± 0.23	0.965

*Different letter superscripts in the same row indicate significant differences (P < 0.05). Values in the same row, either without letter superscripts or the sameletter superscripts indicate no significant difference (P > 0.05). Crude protein levels of 16.0, 14.0, and 12.0% were fed to the control group, group I, and group II, respectively*.

The pH values of the cecum in the 14 and 12% protein groups were reduced by 5.88 and 4.33%, respectively (*P* > 0.05%). A low-protein diet supplemented with amino acids had little effect on the slaughter performance of finishing pigs, but was able to significantly increase the area of the loin eye muscle (*P* < 0.05). Furthermore, a low-protein diet supplemented with amino acid can significantly reduce the heart weight of finishing pigs (*P* < 0.05). When the crude protein level was 14.0%, the lung weight of finishing pigs was significantly lower than that at a protein level of 16.0% (*P* < 0.05). No significant effects were observed on the weights of any other organs (*P* > 0.05).

### Effects of dietary protein levels on the muscle quality of finishing pigs

Table 5 shows that compared with the control group, the shear force in the 14% protein group was increased by 10.66% (*P* > 0.05), whereas the shear stress in the 12% protein group was reduced by 15.85% (*P* < 0.05). In the 14% protein group, the filtration rate was significantly reduced (*P* < 0.05), and the cooking loss was reduced by 2.54% (*P* < 0.05). In the 12% protein group, the marbling score was significantly higher than that in the control group (*P* < 0.05). The drip loss in the 14% protein group was significantly reduced (*P* < 0.05). Compared with the control (16.0% protein group), the shear force of the muscle in finishing pigs was significantly reduced when the crude protein level was 12.0%, and the water loss rate and cooking loss were significantly reduced when the crude protein level was 14.0%. Therefore, a low-protein diet supplemented with amino acids could improve the marbling pattern and quality of meat.

**Table 5:** Effect of dietary protein level on routine indexes of meat quality in finishing pigs.

	**Control group**	**Group I**	**Group II**	***P*-value**
Shear force/*N*	42.40 ± 2.81^a^	46.92 ± 6.84^a^	35.68 ± 5.95^b^	<0.001
Filtration rate/%	6.85 ± 1.50^a^	4.14 ± 1.04^b^	7.04 ± 3.00^a^	0.008
Cooking loss/%	35.82 ± 5.24	34.91 ± 1.81	35.75 ± 1.47	0.809
Marbling score	3.03 ± 0.25^b^	3.11 ± 0.21^ab^	3.30 ± 0.15^a^	0.027
Drip loss/%	3.34 ± 0.26^a^	3.00 ± 0.19^b^	3.22 ± 0.19^a^	0.008
L_45 min_	40.52 ± 2.40^a^	36.75 ± 1.30^b^	40.16 ± 2.76^a^	0.003
L_24 h_	47.89 ± 2.40^a^	40.55 ± 2.38^c^	44.72 ± 3.88^b^	<0.001
a_45 min_	4.32 ± 3.12	4.65 ± 2.28	3.42 ± 1.63	0.550
a_24h_	7.98 ± 0.26^a^	8.02 ± 3.23^a^	5.27 ± 2.26^b^	0.021
b_45 min_	12.57 ± 1.40^a^	7.82 ± 0.92^c^	9.80 ± 1.10^b^	<0.001
b_24 h_	16.48 ± 2.57^a^	14.00 ± 0.77^b^	12.74 ± 2.08^b^	0.002
pH_45 min_	6.19 ± 0.05	6.25 ± 0.02	6.19 ± 0.19	0.455
pH_24 h_	5.71 ± 0.15	5.81 ± 0.14	5.78 ± 0.12	0.300
pH_48 h_	5.53 ± 0.06	5.54 ± 0.03	5.55 ± 0.09	0.678
pH_72 h_	5.50 ± 0.03	5.52 ± 0.06	5.52 ± 0.09	0.769

Different letter superscripts in the same row indicate significant differences (P < 0.05). Values in the same row, either without letter superscripts or the same letter superscripts indicate no significant difference (P > 0.05). Crude protein levels of 16.0, 14.0, and 12.0% were fed to the control group, group I, and group II, respectively. L^*^, lightness value after the specified time; a^*^, redness value after specified time; b^*^, yellowness value after specified time.

Compared with the control group, incarnadine L_45min_ levels in the 14% protein group were reduced by 9.30% (*P* > 0.05). The incarnadine L_24h_ levels in both the 14 and 12% protein groups were reduced by 15.33% (*P* < 0.05) and 6.62% (*P* < 0.05), respectively. The incarnadine b_45min_ values were reduced by 37.79 and 22.04% in the 14% protein group and 12% protein group, respectively (*P* < 0.05). Furthermore, both the 14 and 12% protein groups showed reductions in incarnadine b_24h_ values by 15.05 and 22.69, respectively (*P* < 0.05).

Based on the pH curve, at 24, 48, and 72 h after slaughter, both the 14 and 12% protein groups showed a slower decline in pH than the control group (fed 16% protein). These findings show that less crude protein in the diet can cause muscle pH levels to decline more slowly. The low-protein diet supplemented with amino acids was able to reduce the luminance (L~^*^value) and yellowness (b~^*^value) of meat, slow the decline in pH, and improve meat quality.

### Effects of dietary protein level on muscle nutrients and inosine content in finishing pigs

Compared with the control group, crude fat in the 14% protein group was 21.05% lower; and in the 12% protein group (*P* < 0.05), crude fat was 8.29% lower (*P* > 0.05) (Table 6). However, muscle moisture, crude protein, crude ash, and inosinic acid content showed no significant differences among treatment groups (*P* > 0.05). Thus, a low-protein diet supplemented with amino acids can reduce the crude fat content of muscle, but has no significant effect on other nutrients in muscle.

**Table 6:** Effect of dietary protein level on muscle nutrient contents and levels of inosinic acid in finishing pigs (fresh meat).

	**Control group**	**Group I**	**Group II**	***P*-value**
Moisture/%	71.28 ± 2.64	72.53 ± 0.32	72.60 ± 1.92	0.633
CP/%	22.95 ± 0.25	22.93 ± 0.45	22.83 ± 1.10	0.954
EE/%	7.60 ± 0.10^a^	6.00 ± 0.44^b^	6.97 ± 0.81*a*^b^	0.021
Ash/%	1.19 ± 0.04	1.24 ± 0.07	1.33 ± 0.18	0.131
IMP/(g/kg)	2.69 ± 0.06	2.73 ± 0.28	2.70 ± 0.18	0.965

*Different letter superscripts in the same row indicate significant differences (P < 0.05). Values in the same row, either without letter superscripts or the same letter superscripts indicate no significant difference (P > 0.05). Crude protein levels of 16.0, 14.0, and 12.0% were fed to the control group, group I, and group II, respectively. CP, crude protein; EE, ether extract*.

### Effects of dietary protein level on muscle amino acids of finishing pigs

Compared with the control group, the EAA levels in muscle were increased by 3.32 and 1.95% in the 14 and 12% protein groups, respectively (*P* > 0.05) (Table 7). Furthermore, EAA/TAA values were significantly increased by 3.40 and 1.62%, in the 14% protein and 12% protein groups, respectively (*P* < 0.05). In the 14% protein group, cystine levels were significantly reduced (*P* < 0.05), whereas the low-protein diet with supplemented amino acids showed no significant effects on any of the other 16 amino acids (*P* > 0.05). Glutamate levels and Glu/TAA values in the experimental groups showed an increasing trend, but this difference was not significant (*P* > 0.05). These results show that a low-protein diet supplemented with amino acids could increase EAA and glutamate levels, and thereby improve the quality and flavor of meat.

**Table 7:** Effects of dietary protein level on amino acids in muscle of finishing pigs (fresh meat).

	**Control group**	**Group I**	**Group II**	***P*-value**
Aspartic acid (g/kg)^#^	19.64 ± 0.79	19.55 ± 0.49	19.38 ± 1.07	0.915
Glutamate (g/kg)^#^	26.92 ± 0.80	27.18 ± 0.72	27.10 ± 1.36	0.947
Glycine (g/kg)^#^	8.53 ± 0.16	8.21 ± 0.56	8.08 ± 0.43	0.387
Arginine (g/kg)^#^	14.77 ± 0.44	14.76 ± 0.52	14.79 ± 1.03	0.925
Serine(g/kg)	8.93 ± 0.36	8.59 ± 0.24	8.43 ± 0.38	0.251
Histidine (g/kg)	14.69 ± 1.18	14.36 ± 0.87	14.46 ± 1.77	0.944
Proline (g/kg)	8.97 ± 0.24	8.67 ± 0.41	8.67 ± 0.43	0.565
Alanine (g/kg)	20.38 ± 0.75	20.13 ± 0.65	19.93 ± 0.96	0.745
Threonine (g/kg)^*^	8.01 ± 0.31	7.91 ± 0.17	7.72 ± 0.28	0.463
Tyrosine (g/kg)^*^	9.85 ± 0.39	9.99 ± 0.22	9.90 ± 0.44	0.783
Valine (g/kg)^*^	9.23 ± 0.29	9.82 ± 0.31	9.71 ± 0.51	0.211
Methionine (g/kg)^*^	4.69 ± 0.13	4.80 ± 0.10	4.50 ± 0.42	0.408
Cysteine(g/kg)^*^	10.50 ± 0.11^a^	8.99 ± 0.96^b^	11.70 ± 0.58^a^	0.006
Isoleucine (g/kg)^*^	9.73 ± 0.24^b^	10.68 ± 0.29^a^	10.54 ± 0.58^ab^	0.049
Leucine (g/kg)^*^	17.47 ± 0.64	17.68 ± 0.46	17.60 ± 0.82	0.887
Phenylalanine (g/kg)^*^	8.69 ± 0.29	8.84 ± 0.22	8.77 ± 0.42	0.838
Lysine(g/kg)^*^	20.21 ± 0.55	20.87 ± 0.53	20.70 ± 1.16	0.598
Total AA (g/kg)	221.19 ± 7.64	221.03 ± 7.31	221.97 ± 11.75	0.987
Essential AA (g/kg)	78.01 ± 2.43	80.60 ± 2.03	79.53 ± 3.80	0.571
Flavor AA(g/kg)	69.86 ± 2.19	69.70 ± 2.09	69.34 ± 3.87	0.969
Sweet AA (g/kg)	75.02 ± 2.36	74.38 ± 2.40	73.53 ± 3.61	0.815
Sour AA (g/kg)	61.25 ± 2.76	61.09 ± 2.06	60.94 ± 4.19	0.989
Bitter amino acids(g/kg)	89.11 ± 3.59	90.93 ± 2.91	90.25 ± 5.53	0.858
Glutamate/total AA (%)	12.17 ± 0.06	12.30 ± 0.19	12.21 ± 0.04	0.424
Essential AA/total AA (%)	35.27 ± 0.12^c^	36.47 ± 0.28^a^	35.84 ± 0.31^b^	0.002
Flavor AA/total AA (%)	31.59 ± 0.11^a^	31.54 ± 0.10^a^	31.24 ± 0.14^b^	0.020

# *,umami amino acid;*

* *,essential amino acid. Umami amino acids included glutamate, arginine, aspartic acid, and glycine. Sweet amino acids included glycine, serine, threonine, lysine, proline, and alanine. Acid amino acids included glutamate, aspartic acid, and histidine. Bitter amino acids included histidine, methionine, valine, arginine, leucine, phenylalanine, tryptophan, isoleucine, and tyrosine. Different letter superscripts in the same row indicate significant differences (P < 0.05). Values in the same row, either without letter superscripts or the same letter superscripts indicate no significant difference (P > 0.05). Crude protein levels of 16.0, 14.0, and 12.0% were fed to the control group, group I, and group II, respectively*.

The amino acid values in Table 8 shows that in comparison with the control group, the 14% protein group showed significantly reduced methionine + cystine values in muscle (*P* < 0.05). The methionine + cystine values in the 12% protein group were increased by 6.68% (*P* > 0.05). Isoleucine values in the 14 and 12% protein groups were increased by 9.74 and 8.22%, respectively (*P* > 0.05). Essential amino acid values in the 14 and 12% protein groups were increased by 3.32 and 1.93%, respectively (*P* > 0.05). Therefore, a low-protein diet supplemented with amino acids can improve the EAA values, based on the Food and Agriculture Organization of the United Nations/World Health Organization (FAO/WHO) recommendations for finishing pigs.

**Table 8:** Effect of dietary protein level on essential amino acid levels in the muscle of finishing pigs.

	**Control group**	**Group I**	**Group II**	***P*-value**
Threonine	20.01 ± 0.77	19.77 ± 0.43	19.30 ± 0.70	0.458
Valine	18.45 ± 0.57	19.64 ± 0.62	19.42 ± 1.02	0.212
Methionine + cysteine	43.39 ± 0.69^a^	39.41 ± 3.01^b^	46.29 ± 0.69^a^	0.011
Isoleucine	24.34 ± 0.60	26.71 ± 0.72	26.34 ± 1.45	0.061
Leucine	24.95 ± 0.90	25.25 ± 0.66	25.13 ± 1.17	0.911
Phenylalanine +tyrosine	30.90 ± 1.14	31.38 ± 0.71	31.12 ± 1.41	0.853
Lysine	36.75 ± 1.00	37.95 ± 0.96	37.64 ± 2.12	0.612
Essential AA	22.29 ± 0.69	23.03 ± 0.58	22.72 ± 1.08	0.542

*AA, amino acid. Different letter superscripts in the same row indicate significant differences (P < 0.05). Values in the same row, either without letter superscripts or the same letter superscripts indicate no significant difference (P > 0.05). Crude protein levels of 16.0, 14.0, and 12.0% were fed to the control group, group I, and group II, respectively*.

### Effects of dietary protein levels on muscle fatty acids in finishing pigs

Compared with the control group, the 14 and 12% protein groups showed significantly reduced levels of myristic acid and 15 carbonic acids (*P* < 0.05) (Table 9). In the 14% protein group, the palmitic acid content was significantly reduced (*P* < 0.05). However, no significant difference was observed in the 12% protein group (*P* > 0.05). Fatty acids in both the 14% protein and 12% protein groups were significantly reduced (*P* < 0.05). Oleic acid content was significantly reduced in the 14% protein group (*P* < 0.05); however, the 12% protein group showed no significant difference (*P* > 0.05). In the 14% protein group, linolenic acid content was significantly reduced (*P* < 0.05); however, an increasing trend was noted in the 12% protein group (*P* > 0.05).

**Table 9:** Effects of dietary protein level on fatty acids in the muscle of finishing pigs (fresh meat).

	**Control group**	**Group I**	**Group II**	***P*-value**
**SFA (mg/kg)**				
C10:0	102.50 ± 22.50	151.33 ± 63.31	86.00 ± 14.11	0.187
C12:0	80.00 ± 20.07	52.00 ± 5.00	62.00 ± 9.00	0.085
C14:0	1237.50 ± 47.50^a^	798.33 ± 114.94^b^	975.00 ± 119.55^b^	0.004
C15:0	30.00 ± 5.00^a^	16.50 ± 0.50^b^	12.83 ± 0.76^b^	<0.001
C16:0	19619.00 ± 49.00^a^	14858.33 ± 1 351.01^b^	17553.33 ± 2276.47^ab^	0.022
C17:0	176.50 ± 6.50	135.67 ± 45.46	157.00 ± 61.99	0.553
C18:0	8423.50 ± 780.50	7044.00 ± 365.59	8448.33 ± 1649.02	0.278
C20:0	171.50 ± 9.50	164.00 ± 41.58	135.67 ± 32.65	0.364
**MUFA (mg/kg)**				
C16:1	3236.50 ± 358.50^a^	1998.33 ± 383.75^b^	2513.67 ± 187.20^b^	0.005
C18:1n9c	32869.00 ± 551.00^a^	26174.33 ± 3676.21^b^	30264.33 ± 1532.27^ab^	0.041
C20:1	198.00 ± 34.00	160.33 ± 14.84	160.33 ± 67.28	0.522
C22:1n9	47.00 ± 0.00	43.00 ± 1.00	53.50 ± 12.50	0.263
**PUFA (mg/kg)**				
C18:2n6c	4952.50 ± 545.50	4012.67 ± 700.71	4371.00 ± 2022.26	0.662
C18:3n6	36.50 ± 6.50^ab^	20.50 ± 0.50^b^	50.33 ± 15.37^a^	0.021
C18:3n3	533.50 ± 27.50	537.67 ± 120.40	539.00 ± 88.39	0.989
C20:2	193.00 ± 8.00	181.67 ± 37.00	184.00 ± 62.75	0.939
C20:3n3	45.50 ± 6.50^b^	116.33 ± 30.99^a^	81.67 ± 22.01^ab^	0.024
C20:3n6	105.00 ± 16.00	162.67 ± 37.23	137.67 ± 55.90	0.278
C20:4n6	463.00 ± 100.00	681.67 ± 125.03	785.67 ± 373.55	0.298
C22:6n3	29.50 ± 1.50^b^	20.00 ± 1.00^b^	48.00 ± 12.00^a^	0.005
TFA	72549.50 ± 943.21^a^	57329.33 ± 4141.22^b^	66619.33 ± 7726.68^ab^	0.022
SFA/TFA/%	41.14 ± 1.44	40.50 ± 0.48	41.06 ± 1.98	0.765
UFA/TFA/%	58.86 ± 1.44	59.50 ± 0.48	58.94 ± 1.98	0.823
MUFA/TFA/%	50.10 ± 0.65	49.37 ± 3.65	49.79 ± 3.53	0.912
PUFA/TFA/%	8.76 ± 0.79	10.12 ± 3.31	9.15 ± 2.98	0.811
PUFA/SFA/%	21.35 ± 2.67	24.95 ± 7.94	22.32 ± 7.24	0.773

*SFA, saturated fatty acids; MUFA, monounsaturated fatty acids; PUFA, polyunsaturated fatty acids; UFA, unsaturated fatty acids; MUFA, monounsaturated fatty acids; TFA, total fatty acids. Different letter superscripts in the same row indicate significant differences (P < 0.05). Values in the same row, either without letter superscripts or the same letter superscripts indicate no significant difference (P > 0.05). Crude protein levels of 16.0, 14.0, and 12.0% were fed to the control group, group I, and group II, respectively*.

In the 14% protein group, the levels of 20 carbon triene fatty acids were significantly increased (*P* < 0.05). In the 12% protein group, levels of docosahexaenoic acid were significantly increased (*P* < 0.05). In addition, TFA levels in the 14% protein group were significantly reduced (*P* < 0.05), but showed no significant difference in the 12% protein group (*P* > 0.05). In the 14% protein group, SFA/TFA values were reduced by 1.56% (*P* > 0.05). Furthermore, unsaturated fatty acid (UFA)/TFA values in the 14% protein group were increased by 1.09% (*P* > 0.05). The PUFA/TFA values in the 14% protein and 12% protein groups were increased by 15.53 and 4.45%, respectively (*P* > 0.05). Furthermore, the PUFA/SFA values in the 14% protein and 12% protein groups were 16.86 and 4.54% higher than the control group, respectively (*P* > 0.05). These results show that a low-protein diet supplemented with amino acids could reduce the SFA content in the muscles of finishing pigs, and increase UFA/TFA and PUFA/TFA values, reduce SFA/TFA values, and improve the quality and flavor of pork.

### The effect of dietary protein level on the types and relative levels of volatile compounds in the muscle of finishing pigs

Tables 10, 11 show that the baseline numbers of compounds in the muscle of finishing pigs were reflected by the values in the control group. A total of 197 volatile compounds were detected in the control group, 206 were detected in the 14% protein group and 197 were detected in the 12% protein group. Among the control, 14% protein and 12% protein groups, 18, 24, and 27 aldehyde compounds, respectively were detected. A reduction in dietary crude protein levels increased the types of aldehyde compounds in the muscle. The relative levels of aldehyde compounds in the 12% protein group were increased by 31.60%, compared with the control group (*P* > 0.05).

**Table 10:** Effects of dietary protein level on volatile compounds in the muscle of finishing pigs.

**Chemical**	**Control group**	**Group I**	**Group II**
Aldehyde compounds	18	24	27
Alcohol compounds	25	33	26
Ester compounds	46	38	15
Ketone compounds	11	10	16
Hydrocarbons	54	45	66
Other compounds	43	56	47
Total compounds	197	206	197

*Crude protein levels of 16.0, 14.0, and 12.0% were fed to the control group, group I, and group II, respectively*.

**Table 11:** Effects of dietary protein level on the relative amount of volatile compounds in the muscle of finishing pigs (%).

**Chemical**	**Control group**	**Group I**	**Group II**	***P*-value**
Aldehyde compound	34.34 ± 11.10	33.32 ± 6.46	45.19 ± 13.75	0.388
Alcohol compound	11.30 ± 2.73	12.55 ± 2.85	13.57 ± 0.98	0.526
Ester compounds	23.92 ± 6.24^a^	33.10 ± 8.84^a^	2.36 ± 2.19^b^	<0.001
Ketone compounds	5.11 ± 0.81^b^	4.75 ± 0.73^b^	8.81 ± 1.10^a^	0.002
Hydrocarbon	12.21 ± 5.55	6.23 ± 4.35	13.50 ± 5.99	0.276
Other compounds	13.12 ± 3.09	10.06 ± 6.87	16.53 ± 5.58	0.388

*Different letter superscripts in the same row indicate significant differences (P < 0.05). Values in the same row, either without letter superscripts or the same letter superscripts indicate no significant difference (P > 0.05). Crude protein levels of 16.0, 14.0, and 12.0% were fed to the control group, group I and group II respectively*.

A total of 25, 33, and 26 alcohol compounds were detected in the control, 14% protein and 12% protein groups, respectively. A reduction in protein levels increased the types of alcohol compounds in the muscle. The relative contents of the 14 and 12% protein groups were increased by 11.06 and 20.09% respectively (*P* > 0.05). A total of 46, 38, and 15 ester compounds were detected in the control, 14% protein and 12% protein groups, respectively. A reduction in protein levels reduced the types of ester compounds in the muscle.

The relative contents of the 14% protein group were increased by 38.38% compared with the control group (*P* > 0.05), while the relative contents of the 12% protein group were significantly reduced (*P* < 0.05). A total of 11, 10, and 16 ketone compounds were detected in the control, 14% protein and 12% protein groups, respectively. As the protein levels were reduced, the type and relative levels of ketone compounds in the 14% protein group were also reduced (*P* > 0.05), but significantly increased in the 12% protein group (*P* < 0.05). A total of 54, 45, and 66 types of hydrocarbons were detected in the control, 14% protein and 12% protein groups, respectively. As the dietary protein level was reduced, the relative levels of hydrocarbons in the muscles were first reduced and subsequently increased (*P* > 0.05). A total of 43, 56, and 47 other compounds were detected in the control, 14% protein and 12% protein groups, respectively. A reduction in protein levels increased the levels of other types of compounds, the relative contents of which were first reduced and then increased (*P* > 0.05).

### The effect of dietary protein levels on the relative levels of major volatile compounds in the muscle of finishing pigs

Table 12 shows that a low-protein diet supplemented with amino acids can significantly increase the hexanal content in the muscle of finishing pigs (*P* < 0.05). Neither E-2-heptenal nor E-2-octenal was detected in the control group. However, these compounds were detected in both the 14% protein and 12% protein groups. The levels of E-2-heptenal and E-2-octenal in the 12% protein group were higher than those in the 14% protein group. These findings indicate that reducing protein levels could increase the levels of E-2-heptenal and E-2-octenal.

**Table 12:** Effects of dietary protein level on the relative amounts of major volatile compounds in the muscle of finishing pigs (%).

**Chemical compound**	**Control group**	**Group I**	**Group II**	***P*-value**
Hexanal	9.96 ± 1.82^b^	19.39 ± 1.89^a^	16.93 ± 2.74^a^	0.004
Heptanal	1.49 ± 0.96	1.00 ± 0.13	1.35 ± 0.24	0.576
3-(Methylthio)-propanal	0.21 ± 0.19	0.21 ± 0.19	0.11 ± 0.04	0.626
(E)-2-Heptenal	–	0.24 ± 0.04^b^	0.66 ± 0.08^a^	<0.001
Benzaldehyde	5.07 ± 2.30	3.62 ± 2.48	3.33 ± 0.43	0.533
(E)-2-Octenal	–	0.68 ± 0.52	0.84 ± 0.74	0.187
Benzeneacetaldehyde	0.46 ± 0.33	0.21 ± 0.06	0.40 ± 0.09	0.365
Nonanal	5.39 ± 2.83	3.45 ± 3.17	7.10 ± 1.76	0.289
(E)-2-Decenal	0.95 ± 0.86	0.51 ± 0.13	1.41 ± 1.11	0.419
Tetradecanal	0.68 ± 0.08^b^	1.32 ± 0.22^b^	3.99 ± 2.42^a^	0.046
1-Pentanol	0.47 ± 0.34	0.29 ± 0.07	0.45 ± 0.39	0.689
1-Hexanol	0.25 ± 0.13	0.25 ± 0.19	0.19 ± 0.04	0.769
1-Heptanol	0.39 ± 0.09	0.32 ± 0.09	0.45 ± 0.13	0.356
1-Octen-3-ol	2.47 ± 0.47^b^	3.89 ± 0.90^a^	4.08 ± 0.27^a^	0.026
1-Octanol	1.16 ± 0.38	0.69 ± 0.56	1.19 ± 0.32	0.316
Thiocyanic acid carbazol-3,6-diyl ester	0.43 ± 0.23	0.21 ± 0.12	0.23 ± 0.22	0.335
2-Heptanone	0.16 ± 0.04	0.24 ± 0.07	0.28 ± 0.07	0.113
2,3-Octanedione	0.10 ± 0.03^b^	0.38 ± 0.07^b^	2.10 ± 0.42^a^	<0.001
Limonene	0.21 ± 0.13^ab^	0.34 ± 0.04^a^	0.12 ± 0.04^b^	0.048
Furan, 2-pentyl-	1.13 ± 0.52^b^	0.77 ± 0.17^b^	2.01 ± 0.15^a^	0.005
7H-Dibenzo[b,g]carbazole, 7-methyl-	1.79 ± 0.53	2.03 ± 0.31	3.19 ± 2.60	0.532
n-Hexadecanoic acid	1.50 ± 1.26	0.77 ± 0.52	2.26 ± 1.26	0.289

*Different letter superscripts in the same row indicate significant differences (P < 0.05). Values in the same row, either without letter superscripts or the same letter superscripts indicate no significant difference (P > 0.05). Crude protein levels of 16.0, 14.0, and 12.0% were fed to the control group, group I and group II, respectively*.

In both the 14% protein and 12% protein groups, levels of benzaldehyde and phenylacetaldehyde were lower than those in the control group (*P* > 0.05). Compared with the control group, the levels of 1-octen-3ol in the 14% protein and 12% protein groups were significantly increased (*P* < 0.05). Furthermore, in both the 14% protein and 12% protein groups, levels of 2-heptanone and 2,3-octanone were increased compared with the control group. Levels of 2,3-octanedione in the 12% protein group were significantly increased compared with both the control group and the 14% protein group (*P* < 0.05). Compared with the control group, 2-pentylfuran levels were reduced in the 14% protein group, but significantly increased in the 12% protein group (*P* < 0.05). Compared with the control group, levels of 7-methyl-7H-dibenzo [b,g]carbazole in both the 14% protein and 12% protein groups were increased.

## Discussion

### Effects of dietary protein level on the growth performance of finishing pigs

Studies have shown that the growth performance of finishing pigs is not affected by reductions in dietary protein levels with appropriate supplementation of amino acids (17). They Adding 0.49% alanine and 1% tyrosine to the diet of duchangda three way hybrid finishing pigs with 12.52% protein level was beneficial to the growth performance of finishing pigs, but it would not significantly affect the ADFI, ADG and ratio of feed to weight (18). The crude protein level of growing pigs was reduced, and the addition of lysine had no significant effect on the performance of growing pigs (19).

When the dietary protein level was reduced by 1–2% on the basis of 14.8%, there was no significant effect on the growth performance and apparent digestibility of nutrients of finishing pigs (20). The results of the current study are consistent with those of the aforementioned studies. The current study found that 14.0% crude protein could increase the average daily gain of finishing pigs and reduce the feed-to-weight ratio, compared with the control (16.0% crude protein); however the differences were not significant. The low-protein diets supplemented with amino acids had no significant effects on growth performance in finishing pigs.

### Effects of dietary protein level on slaughter performance, organ weight, and intestinal pH of finishing pigs

Whether dietary protein levels can alter the carcass and meat quality is controversial, but most scholars believe that the low-protein diet has no significant effect on the slaughter performance of finishing pigs after balancing the nutritional requirements of supplementing the essential amino acids of the diet, with back fat and skin thickness reduced by 15.6 and 11.5%, respectively, while the proportion of lean meat increased (21). Chen et al. fed (12%, three different protein levels, 14%, 16%) to DuYueba ternary hybrid pigs and showed that different protein levels did not significantly affect the slaughter performance of finishing pigs (22). However, the carcass weight and lean meat percentage of the finishing pigs in their 14 and 16% protein groups were higher than those in the 12% protein group. Furthermore, they reported that the back fat thickness and fat percentage were reduced with increasing protein level.

In the present study, feeding a low-protein diet with supplemented amino acids was able to reduce the back fat and skin thickness of finishing pigs. Differences in the results of various studies could be attributed to differences in the selection of pig breeds. Yang et al. found that appropriate reductions in dietary protein levels and supplementation of amino acids had no significant effect on dressing percentage, loin eye muscle area, lean meat percentage and fat percentage (23). Sobotka et al. found that the lean meat percentage of finishing pigs tended to increase after being fed a low-protein diet and reported an increase in the loin eye muscle area of 6.80% (24).

Zhang et al. reported that the dressing percentage of their low-protein group was increased by 1.33% and the loin eye muscle area was increased by 5.72% (25). The current study showed that reducing the levels of dietary crude protein can increase the loin eye muscle area of finishing pigs. The results of Chen et al. (7) showed that low-protein diets have no significant effects on the weights of internal organs of finishing pigs. The current study found that a low-protein diet supplemented with amino acids can significantly reduce the weights of the heart and lungs but has no significant effects on the weights of other organs of finishing pigs.

### Effects of dietary protein level on the quality of meat in finishing pigs

The results of studies on the effects of dietary crude protein levels on pork quality show wide variations. Zhu et al. found that low-protein diets supplemented with amino acids can significantly reduce the shear force of the longissimus dorsi of finishing pigs, without any significant effects on muscle pH, meat color, L^*^, a^*^ or b^*^ values, cooking loss, and drip loss (26). Zhang et al. reported no significant differences in flesh color (L^*^, a^*^ and b^*^), or muscle pH between the normal protein group and the low-protein group (27). Goerl et al. reported that low-protein diets can increase marbling score and improve muscle tenderness (28). Bidner et al. found that dietary protein levels had no significant effect on muscle color, pH, or drip loss (29). Ruusunen et al. found that feeding low-protein diets can reduce the pH of muscle in finishing pigs within 45 min and increase drip loss but show no significant effects on muscle pH after 24 h (30).

Li et al. fed finishing pigs diets with crude protein levels of 14 and 10% that low-protein diets showed no significant effects on the color of the longissimus dorsi muscle, pH_45min_, pH_24h_, and drip loss (6). Zhang et al. found that the drip loss of finishing pigs fed a low-protein diet was significantly higher than that of pigs fed a diet with normal protein levels (27); however, neither the pH_45min_ nor pH_24h_ showed significant differences from the normal protein group. The current study showed that when the dietary crude protein level was 14.0% the muscle dehydration rate and drip loss were significantly reduced compared with the 16.0% protein group. When the protein level was 12.0% the reduction in muscle shear force and the improvement in muscle marbling score were both significant compared with the 16.0% protein group. The 12.0 and 14.0% protein groups showed significantly reduced muscle color L_24_, b_45min_ and b_24h_ which improved the muscle quality of finishing pigs. However, neither the 12.0% nor 14.0% protein groups showed any significant effects on muscle pH.

### Effects of dietary protein level on the nutritional composition and inosinic acid levels in the muscle of finishing pigs

The nutrients contained in the meat of pigs determine the nutritional value of pork. Hu et al. found that the intermuscular fat contents of pigs fed different levels of crude protein were decreased with as protein level was increased while the crude protein content of muscle was increased with increasing dietary protein level (31). These findings indicated that low-protein diets can reduce the fat content and crude protein content of muscle. Huo et al. established three protein levels (11.96, 13.04, and 14.16%, respectively) in three groups, and reported no significant differences in the dry matter, crude ash, crude protein, or inosinic acid levels in any group; however, the crude fat and cholesterol levels in the low-protein group were significantly reduced (32).

(33) found that with increasing dietary protein levels, the crude protein content in muscle is also increased, but the content of crude fat and inosinic acid shows a decline (33). Li et al. reported that dietary protein levels had no significant effects on inosinic acid levels in the muscle of different breeds of chickens (34); however, inosinic acid levels were increased with reduced dietary protein levels. The current study showed that when the crude protein level in the diet was 14.0%, the crude fat content in the muscle was significantly reduced, compared with the 16.0% protein group (control). The crude protein level in the diet had no significant effects on the water content of muscle, crude ash, crude protein, and inosinic acid.

Amino acids are the basic units of protein molecules. The amino acid levels and composition have an important influence on meat quality and flavor. Among the various amino acids, EAA (lysine, phenylalanine, methionine, threonine, tryptophan, isoleucine, leucine and valine) and umami amino acids (glutamic acid, arginine, aspartic acidc and glycine) are often used as indicators in the evaluation of muscle quality and flavor (35). Chen reported that dietary protein levels had no significant effects on the amino acid content of Duyueba three-way crossbred pigs, and differences in the levels of EAA and savory amino acids were not significant (22).

The current study found that low-protein diets supplemented with amino acids can significantly increase EAA/TAA values and increase the total amount of EAA. Compared with the 16.0% protein group when the crude protein level was 14.0%, no significant effects were observed on FAA/TAA values; however, FAA/TAA values were significantly decreased when the crude protein level was 12.0%. Glutamate plays an important role in improving the flavor of pork because of its buffering effect on unpleasant odors (36). The current study found that a reduced dietary crude protein levels can also increase the levels of glutamate in the muscle which can improve the flavor of pork. The amino acid score can be used as an index to determine the nutritional value of food. The higher the amino acid score, the better the nutritional value of the food (37). The current study showed that low-protein diets supplemented with amino acids can improve the levels of EAA in the muscle of finishing pig. These findings indicate that appropriate reductions in dietary crude protein levels can increase the nutritional value of pork and improve meat quality.

### Effects of dietary protein levels on fatty acids in the muscle of finishing pigs

Fatty acids include saturated fatty acids and unsaturated fatty acids. Excessive intake of saturated fatty acids in humans can cause diseases such as arteriosclerosis. Unsaturated fatty acids can have positive effects on human health, such as anti-cancer, lipid-lowering effects, and the prevention of cardiovascular disease (38–40). Huo et al. found that low-protein diets can significantly reduce linolenic acid levels in muscle and increase arachidic acid levels, but show no significant effects on other fatty acids (32).

The current study showed that low-protein diets supplemented with amino acids can reduce linolenic acid levels in muscle. However, much lower dietary protein levels can increase linolenic acid levels in muscle, while low-protein diets can increase arachidonic acid levels. These results are consistent with those of the study of Zhou et al. (41), who reported that low-protein diets can reduce the stearic acid, palmitic acid, and linoleic acid levels, and increase oleic acid levels in the longissimus dorsi muscle. The current study showed that low-protein diets can also reduce stearic acid, palmitic acid, and linoleic acid levels, as well as oleic acid levels in muscle. The differences in results between studies could be attributed to differences in the selected breeds of pigs.

Teye et al. found that low-protein diets have a tendency to reduce palmitic acid and stearic acid levels in muscle, which is consistent with the results of the current study (42). Furthermore, low-protein diets have no significant effects on the levels of SFA, MUFA, and PUFA. Martinezaispuro et al. reported that SFA and MUFA levels in a low-protein group were higher than those in a comparatively high-protein group, while the PUFA levels were reduced. However, the results of the current study differ from those findings (43).

The current study showed that low-protein diets supplemented with amino acids reduced SFA/TFA and MUFA/TFA values, but increased PUFA/TFA values. The differences in results could be attributed to differences in the pig breeds selected, diet composition, and feeding environment. The PUFA/SFA values are also commonly used as meat quality evaluation indicators. Regarding human nutrition, meat PUFA/SFA values should be 0.45 or slightly higher (44), and WHO recommends PUFA/SFA levels >0.4 (45). The current study showed that a low-protein diet supplemented with amino acids could improve the PUFA/SFA values in the muscle of finishing pigs and improve meat quality.

### Effects of dietary protein level on the types and relative content of volatile compounds in the muscle of finishing pigs

Aldehydes are the most important volatile components in pork, and they have the greatest effects on pork flavor. The odor threshold of aldehyde compounds is low, and they have strong recognizable odors (46). The current study showed that compared with the 16.0% protein group, the dietary crude protein levels of 14.0 and 12.0% increased the types of aldehyde compounds in the muscles of finishing pigs. In addition, the crude protein level of 12.0% increased the relative levels of aldehyde compounds.

The alcohol contents in pork is also high, which contributes to its flavor. The current study showed that when the dietary crude protein level was 14.0%, the types of alcohol compounds were more than those of the control diet (with a crude protein level of 16.0%). Furthermore, the relative levels of alcohol compounds in the 14.0 and 12.0% protein groups were higher than those in the 16.0% protein group.

Ester compounds have little effect on the flavor of pork, and no such compoundis known to reflect the characteristic flavor of pork (47). The current study showed that in the 16.0% protein group, levels of ester compounds were higher than those in the 14.0 and 12.0% protein groups. Furthermore, and the relative contents of ester compounds in the 16.0 and 14.0% protein groups were significantly higher than those in the 12.0% protein group.

Most ketone compounds have floral or fruity aromas, and the longer the carbon chain, the stronger the floral characteristics (48). The current study showed that the types and relative levels of ketones in the 12.0% protein group were higher than those in the 16.0% protein group. Hydrocarbons have a high threshold, and therefore have little direct effect on pork flavor. However, hydrocarbons are intermediates of heterocyclic compounds, and still have basic effects on pork flavor (49). The current study showed that as the dietary crude protein level was reduced, the types and relative content of hydrocarbons were first reduced and then subsequently increased.

### Effects of dietary protein level on the relative content of main volatile compounds in the muscle of finishing pigs

Hexanal has a grassy odor, andis one of the most abundant aldehydes in meat (47). Other aldehyde compounds may have different flavors. The current study showed that a reduction in dietary crude protein levels increased the hexanal content in the muscles of finishing pigs, and increased the E-2-heptenal and E-2-octenal levels. Alcohol compounds also play an important role in pork flavor. Among them, levels of 1-octene-3-ol, which has the flavor of cooked mushrooms, were relatively higher in meat, and plays an important role in pork flavor (50). The levels of 2-heptanone and 2,3-octanedione in ketones are relatively high. Furthermore, 2-heptanone has a fruity aroma, and 2,3-octanedione is a unique component of pork that imparts a characteristic aroma (51, 52).

The current study showed that the levels of 2-heptanone and 2,3-octanedione were increased as the protein level was reduced. Furans and nitrogen-containing compounds also play an important role in pork flavor. In addition, 2-pentylfuran has a bean and vegetable flavor (11, 53). The current study showed that as the level of dietary crude protein was reduced, the 2-pentylfuran content was first reduced and then increased. The low-protein diets supplemented with amino acids increased the levels of the nitrogenous compound, 7-methyl-7H-dibenzo[b, g]carbazole. In addition, a low-protein diet supplemented with amino acids can increase the levels of characteristic flavor compounds.

## Conclusion

In conclusion, compared with the existing commercial feed nutrition standards, the dietary protein level of finishing pigs is reduced by 2.0–4.0%, which has no significant impact on the growth performance and slaughtering performance of finishing pigs, but it can improve the slaughtering performance and meat quality to varying degrees, and increase the quantity and content of volatile flavor substances in muscle, which has a positive impact on improving meat quality. Among them, the dietary protein level of 14.0% is the best.

## Data availability statement

The original contributions presented in the study are included in the article/supplementary material, further inquiries can be directed to the corresponding author/s.

## Ethics statement

The animal study was reviewed and approved by Ethics Committee for the Care and Use of Laboratory Animals of Gansu Agricultural University.

## Author contributions

GC and DW designed the study. GC, DW, MC, YL, YC, CS, and YGe performed the experiments and collected samples. DW, SL, YGa, and HH analyzed the data. DW and GC wrote the manuscript. All authors contributed to manuscript revision and read and approved the final version.

## Funding

This study was supported by the National Natural Science Foundation of China effects of different feed protein level on the diversity of intestinal flora and meat quality in pigs (31960665) and Lanzhou Science and Technology Bureau Project livestock and poultry non-anti-healthy flavor livestock product research and development center (2019-4-52; 2020-1-10).

## Conflict of interest

The authors declare that the research was conducted in the absence of any commercial or financial relationships that could be construed as a potential conflict of interest.

## Publisher's note

All claims expressed in this article are solely those of the authors and do not necessarily represent those of their affiliated organizations, or those of the publisher, the editors and the reviewers. Any product that may be evaluated in this article, or claim that may be made by its manufacturer, is not guaranteed or endorsed by the publisher.

## References

[1] Wang Y Zhou J Wang G Cai S Zeng X Qiao S. Advances in low-protein diets for swine. J Anim Sci Biotechnol. (2018) 9:60. 10.1186/s40104-018-0276-7 30034802 PMC6052556

[2] Nancy MH Jonnelle LM Christopher PB Shihshieh H Michael HL Thomas MM. High-lysine corn generated by endosperm-specific suppression of lysine catabolism using RNAi. Plant Biotechnol J. (2010) 5:605–14. 10.1111/j.1467-7652.2007.00265.x 17553105

[3] Gloaguen M Le FN Corrent E Primot Y Milgen J. The use of free amino acids allows formulating very low crude protein diets for piglets. J Anim Sci. (2014) 92:637–44. 10.2527/jas.2013-6514 24398840

[4] Wang B Mi MM Zhang QY Bao N Pan L Zhao Y. Relationship between the amino acid release kinetics of feed proteins and nitrogen balance in finishing pigs. Animal. (2021) 15:100359. 10.1016/j.animal.2021.100359 34536654

[5] Xu Y Chen H Wan K Zhou K Wang Y Li J. Effects of supplementing low-protein diets with sodium dichloroacetate and glucose on growth performance, carcass traits, and meat quality of growing-finishing pigs. J Anim Sci. (2022) 100:skab359. 10.1093/jas/skab359 34865045 PMC8722760

[6] Li N Xie CY Zeng XF Wang DH Qiao SY. Effects of dietary crude protein level and amino acid balance on growth performance, carcass traits and meat quality of finishing pigs. J Anim Nutr. (2018) 30:498–506. 10.3969/j.issn.1006-267x.2018.02.013

[7] Chen J Zhang H Gao H Kang B Chen F Li Y. Effects of dietary supplementation of alpha-ketoglutarate in a low-protein diet on fatty acid composition and lipid metabolism related gene expression in muscles of growing pigs. Animals. (2019) 9:838. 10.3390/ani9100838 31640132 PMC6826391

[8] Wang D Chen GS Chai MJ Wang YF Yao YY Zhang X. Effects of low protein diet on growth performance and meat quality of Du × Min hybrid finishing pigs. Chin J Anim Sci. (2020) 56:146–9. 10.19556/j.0258-7033.20191219-07

[9] Bai XL. Effects of Different Protein Sources of Low Protein Diet on Growth Performance Dynamic Changes of Amino Acids in Small Intestine of Finishing Pigs (Master's thesis). Jilin Agricultural University (2017). Available online at: https://kns.cnki.net/KCMS/detail/detail.aspx?dbname=CMFD201801&filename=1017842924.nh (accessed January 16, 2018).

[10] Wu LY. The Effect of Microecological Preparations Achyranthesbidentata Polysaccharides on Pig Growth Performance Meat Quality (Master's thesis). Changsha: Hunan Agricultural University (2011). Available online at: https://kns.cnki.net/KCMS/detail/detail.aspx?dbname

[11] Chen GS Cai Y Su YY Wang D Pan XL Zhi XJ. Study of meat quality and flavour in different cuts of Duroc?Bamei binary hybrid pigs. Veter Med Sci. (2020) 7:724–34. 10.1002/vms3.409 33326708 PMC8136970

[12] Škrlep M Poklukar K Kress K Vrecl M Fazarinc G Batorek LN. Effect of immunocastration and housing conditions on pig carcass and meat quality traits. Transl Anim Sci. (2020) 4:txaa055. 10.1093/tas/txaa055 32705051 PMC7284115

[13] Liu P Zhang H Mi Z Zhang L Zhang G. Determination of 11 sulfonamides in pork by two-step liquid-liquid extraction-solid phase extraction purification coupled with high performance liquid chromatography-tandem mass spectrometr. Se Pu. (2019) 37:1098–104. Chinese. 10.3724/SP.J.1123.2019.04005 31642289

[14] Wang J Wang H Xue J Yu X Long X Wu X. Phospholipidomics quality evaluation of swimming crabs (*Portunus trituberculatus*) cultured with formulated feed, frozen trash fish, and mixed feed, a non-target approach by HILIC-MS. J Chromatogr B Analyt Technol Biomed Life Sci. (2021) 1179:122845. 10.1016/j.jchromb.2021.122845 34218091

[15] Hoa VB Kyeong SR Nguyen TKL Inho H. Influence of particular breed on meat quality parameters, sensory characteristics, and volatile components. Food Sci Biotechnol. (2013) 22:651–8. 10.1007/s10068-013-0127-4

[16] Chen G Sui Y Chen S. Detection of flavor compounds in longissimus muscle from four hybrid pig breeds of Sus scrofa, Bamei pig, and Large White. Biosci Biotechnol Biochem. (2014) 78:1910–6. 10.1080/09168451.2014.936348 25052201

[17] Zhou J Wang L Zhou J Zeng X Qiao S. Effects of using cassava as an amylopectin source in low protein diets on growth performance, nitrogen efficiency, and postprandial changes in plasma glucose and related hormones concentrations of growing pigs. J Anim Sci. (2021) 99:skab332. 10.1093/jas/skab332 34850908 PMC8722424

[18] Shi BM Bai GD Xu X He W Yang Z Gao F. Effects of adding tyrosine to low protein diets on the growth performance and nitrogen metabolism of fattening pigs. J Northeast Agric Univ. (2019) 50:58–66. 10.19720/j.cnki.issn.1005-9369.2019.6.007

[19] Veira AM Dos Santos LS Campos PHRF Marcal DA Fraga AZ Hauschild L. Effects of sequential feeding with adjustments to dietary amino acid concentration according to the circadian rhythm on the performance, body composition, and nutrient balance of growing-finishing pigs. PLoS ONE. (2021) 16:e0261314. 10.1371/journal.pone.0261314 34941900 PMC8700050

[20] Zeng YX Wang J Ji HF Wang SX Liu H Zhang DY. Effects of low-protein diets on production performance, nitrogen metabolism and blood biochemical indexes of fattening pigs. J Livestock Ecol. (2017) 38:30–6.

[21] Maeda K Yamamoto F Toyoshi M Irie M. Effects of dietary lysine/protein ratio and fat levels on growth performance and meat quality of finishing pigs. Anim Sci J. (2014) 85:427–34. 10.1111/asj.12150 24261827

[22] Chen J. Effects of Dietary Protein Levels on the Growth Performance and Meat Quality Traits of Duyueba Ternary Hybrid Pigs (Master's thesis). Northwest A&F University, Xianyang, China (2016).

[23] Yang Q Zhang SR He X Ma XL Lu DW Zhang HF. Effects of different energy levels in low-protein diets on growth performance and carcass traits of fattening pigs. J Anim Nutr. (2008) 4:371–6. 10.3969/j.issn.1006-267X.2008.04.001

[24] Sobotka W Fiedorowicz-Szatkowska E. The effect of replacing genetically modified soybean meal with 00-rapeseed meal, faba bean and yellow lupine in grower-finisher diets on nutrient digestibility, nitrogen retention, selected blood biochemical parameters and fattening performance of pigs. Animals. (2021) 11:960. 10.3390/ani11040960 33808314 PMC8065711

[25] Zhang GJ Yi XW Lu N Tan ZL. Effects of using net energy system to formulate low-protein diets on the growth performance and carcass quality of growing and fattening pigs. J Anim Nutr. (2010) 22:557–63.

[26] Zhu YP Zhou P Li JL Zhang L Gao F Zhou GH. Effects of adding cysteamine to low-protein amino acid balanced diet on growing pork quality and related gene expression. J Anim Husb Vet Med. (2017) 48:660–8. 10.11843/j.issn.0366-6964.2017.04.009

[27] Zhang SH Chu LC Qiao SY Mao X Zeng X. Effects of dietary leucine supplementation in low crude protein diets on performance, nitrogen balance, whole-body protein turnover, carcass characteristics and meat quality of finishing pigs. Anim Sci J. (2016) 87:911–20. 10.1111/asj.12520 26597995

[28] Goerl KF Eilert SJ Mandigo RW Chen HY Miller PS. Pork characteristics as affected by two populations of swine and six crude protein levels. J Anim Sci. (1995) 73:12:3621–6. 10.2527/1995.73123621x 8655436

[29] Bidner BS Ellis M Witte DP Carr SN McKeith FK. Influence of dietary lysine level,pre-slaughter fasting,andrendementnapole genotype on fresh pork quality. Meat Sci. (2004) 68:53–60. 10.1016/j.meatsci.2003.10.018 22062007

[30] Ruusunen M Partanen K Pösö R Puolannea E. The effect of dietary protein supply on carcass composition,size of organs,muscle properties and meat quality of pigs. Livest Sci. (2006) 107:170–81. 10.1016/j.livsci.2006.09.021

[31] Hu XQ. Effect of Alfalfa Meal and Dietary Crude Protein Level on Pig Carcass and Meat Quality (Master's degree thesis). Southwest University, El Paso, TX, United States (2006).

[32] Huo YJ Zhan JS Yu TS Zhu JP Zhao GQ. Effects of different dietary protein levels on growth performance, meat quality and serum biochemical indexes of Huai pigs. Acta Prata Sinica. (2015) 24:133–41. 10.11686/cyxb2014310

[33] Alonso V Campo Mdel M Provincial L Roncalés P Beltrán JA. Effect of protein level in commercial diets on pork meat quality. Meat Sci. (2010) 85:7–14. 10.1016/j.meatsci.2009.11.015 20374857

[34] Li SY Xu Y Li QH Yu HX Wang YH. Effects of variety and dietary nutrition level on the content of inosinic acid in chicken. China Anim Husb Vet Med. (2007) 10:133–5. 10.3969/j.issn.1671-7236.2007.10.049

[35] Benz JM Tokach MD Dritz SS Nelssen JL Derouchey JM Sulabo RC. Effects of increasing choice white grease in corn- and sorghum-based diets on growth performance, carcass characteristics, and fat quality characteristics of finishing pigs. J Anim Sci. (2011) 89:773–82. 10.2527/jas.2010-3033 21346136

[36] Liu Y Li Y Peng Y He J Xiao D Chen C. Dietary mulberry leaf powder affects growth performance, carcass traits and meat quality in finishing pigs. J Anim Physiol Anim Nutr. (2019) 103:1934–45. 10.1111/jpn.13203 31478262

[37] Berrazaga I Micard V Gueugneau M Walrand S. The role of the anabolic properties of plant- versus animal-based protein sources in supporting muscle mass maintenance: a critical review. Nutrients. (2019) 11:1825. 10.3390/nu11081825 31394788 PMC6723444

[38] Siri-Tarino PW Sun Q Hu FB Krauss RM. Saturated fat, carbohydrate, and cardiovascular disease. Am J Clin Nutr. (2010) 91:502–9. 10.3945/ajcn.2008.26285 20089734 PMC2824150

[39] De Oliveira PA Kovacs C Moreira P Magnoni D Saleh MH Faintuch J. Unsaturated fatty acids improve atherosclerosis markers in obese and overweight non-diabetic elderly patients. Obes Surg. (2017) 27:2663–71. 10.1007/s11695-017-2704-8 28470492

[40] Mukherjee A Kenny HA Lengyel E. Unsaturated fatty acids maintain cancer cell stemness. Cell Stem Cell. (2017) 20:291–2. 10.1016/j.stem.2017.02.008 28257705 PMC5956908

[41] Zhou X Liu Y Zhang L Kong X Li F. Serine-to-glycine ratios in low-protein diets regulate intramuscular fat by affecting lipid metabolism and myofiber type transition in the skeletal muscle of growing-finishing pigs. Anim Nutr. (2021) 7:384–92. 10.1016/j.aninu.2020.08.011 34258426 PMC8245814

[42] Teye GA Sheard PR Whittington FM Nute GR Stewart A Wood JD. Influence of dietary oils and protein level on pork quality. 1 Effects on muscle fatty acid composition, carcass, meat and eating quality. Meat Sci. (2006) 73:157–65. 10.1016/j.meatsci.2005.11.010 22062065

[43] Martinezaispuro M Figueroavelasco JL Zamorazamora V Sancheztorres MT Ortegacerrilla ME Corderomora JL. Effect of fatty acids source on growth performance, carcass characteristics, plasma urea nitrogen concentration, and fatty acid profile in meat of pigs fed standard- or low-protein diets. Spanish J Agric Res. (2012) 10:993–1004. 10.5424/sjar/2012104-323-11

[44] Han D Zhang CH Fauconnier ML Mi S. Characterization and differentiation of boiled pork from Tibetan, Sanmenxia and Duroc × (Landrac × Yorkshire) pigs by volatiles profiling and chemometrics analysis. Food Res Int. (2020) 130:108910. 10.1016/j.foodres.2019.108910 32156356

[45] Xing K Wang K Ao H Chen S Tan Z Wang Y. Comparative adipose transcriptome analysis digs out genes related to fat deposition in two pig breeds. Sci Rep. (2019) 9:12925. 10.1038/s41598-019-49548-5 31501489 PMC6733950

[46] Han S Schroeder EA Hebestreit K Mair WB Brunet A. Mono-unsaturated fatty acids link H3K4me3 modifiers to *C. elegans* lifespan. Nature. (2017) 544:185–90. 10.1038/nature21686 28379943 PMC5391274

[47] Huff-Lonergan E Baas TJ Malek M Dekkers JC Prusa K Rothschild MF. Correlations among selected pork quality traits. J Anim Sci. (2002) 80:617–27. 10.2527/2002.803617x 11892678

[48] Estévez M Morcuende D Ventanas S Cava R. Analysis of volatiles in meat from Iberian pigs and lean pigs after refrigeration and cooking by using SPME-GC-MS. J Agric Food Chem. (2003) 51:3429–35. 10.1021/jf026218h 12744679

[49] Mottram DS. Flavour formation in meat and meat products: a review. Food Chem. (1998) 62:415–24. 10.1016/S0308-8146(98)00076-4

[50] Wood JD Nute GR Richardson RI Whittington FM Southwood O Plastow G. Effects of breed, diet and muscle on fat deposition and eating quality in pigs. Meat Sci. (2004) 67:651–67. 10.1016/j.meatsci.2004.01.007 22061815

[51] Calkins CR Hodgen JM. A fresh look at meat flavor. Meat Sci. (2007) 77:63–80. 10.1016/j.meatsci.2007.04.016 22061397

[52] Ramírez MR Estévez M Morcuende D Cava R. Effect of the type of frying culinary fat on volatile compounds isolated in fried pork loin chops by using SPME-GC-MS. J Agric Food Chem. (2004) 52:7637–43. 10.1021/jf049207s 15675815

[53] Choi JY Shinde P Jin Z Kim JS Chae BJ. Effects of dietary protein level and phase feeding regimen on growth performance, carcass characteristics and pork quality in growing-finishing pigs. J Anima Sci Technol. (2010) 52:205–12. 10.5187/JAST.2010.52.3.205

